# Plasma fatty acid changes following consumption of dietary oils containing n-3, n-6, and n-9 fatty acids at different proportions: preliminary findings of the Canola Oil Multicenter Intervention Trial (COMIT)

**DOI:** 10.1186/1745-6215-15-136

**Published:** 2014-04-23

**Authors:** Vijitha K Senanayake, Shuaihua Pu, David A Jenkins, Benoît Lamarche, Penny M Kris-Etherton, Sheila G West, Jennifer A Fleming, Xiaoran Liu, Cindy E McCrea, Peter J Jones

**Affiliations:** 1 Richardson Centre for Functional Foods and Nutraceuticals, University of Manitoba, 196 Innovation Drive, SmartPark, Winnipeg, MB R3T 6C5, Canada; 2 St. Michaels Hospital, 30 Bond Street, Toronto, ON M5B 1W8, Canada; 3 Institute of Nutrition and Functional Foods, Pavillon des Services, Université Laval, Suite 1705, 2440 Hochelaga Blvd, Québec, QC G1V 0A6, Canada; 4 Department of Nutritional Sciences, Pennsylvania State University, 110 Chandlee Laboratory, University Park, PA 16802, USA; 5 Department of Biobehavioral Health, Pennsylvania State University, 219 Biobehavioral Health Building, University Park, PA 16802, USA; 6 Current address: Phenomenome Discoveries Inc, Saskatoon, Saskatchewan, Canada

**Keywords:** Cardiovascular diseases, Metabolic syndrome, Plasma fatty acids, Serum lipids, Lipoproteins, Canola oil, DHA, Randomized controlled clinical trial

## Abstract

**Background:**

The Canola Oil Multicenter Intervention Trial (COMIT) was a randomized controlled crossover study designed to evaluate the effects of five diets that provided different oils and/or oil blends on cardiovascular disease (CVD) risk factors in individuals with abdominal obesity. The present objective is to report preliminary findings on plasma fatty acid profiles in volunteers with abdominal obesity, following the consumption of diets enriched with n-3, n-6 and n-9 fatty acids.

**Methods:**

COMIT was conducted at three clinical sites, Winnipeg, Manitoba, Canada, Québec City, Québec, Canada and University Park, Pennsylvania, United States. Inclusion criteria were at least one of the followings: waist circumference (≥90 cm for males and ≥84 cm for females), and at least one other criterion: triglycerides ≥1.7 mmol/L, high density lipoprotein cholesterol <1 mmol/L (males) or <1.3 mmol/L (females), blood pressure ≥130 mmHg (systolic) and/or ≥85 mmHg (diastolic), and glucose ≥5.5 mmol/L. Weight-maintaining diets that included shakes with one of the dietary oil blends were provided during each of the five 30-day dietary phases. Dietary phases were separated by four-week washout periods. Treatment oils were canola oil, high oleic canola oil, high oleic canola oil enriched with docosahexaenoic acid (DHA), flax oil and safflower oil blend, and corn oil and safflower oil blend. A per protocol approach with a mixed model analysis was decided to be appropriate for data analysis.

**Results:**

One hundred and seventy volunteers were randomized and 130 completed the study with a dropout rate of 23.5%. The mean plasma total DHA concentrations, which were analyzed among all participants as a measure of adherence, increased by more than 100% in the DHA-enriched phase, compared to other phases, demonstrating excellent dietary adherence.

**Conclusions:**

Recruitment and retention strategies were effective in achieving a sufficient number of participants who completed the study protocol to enable sufficient statistical power to resolve small differences in outcome measures. It is expected that the study will generate important data thereby enhancing our understanding of the effects of n-3, n-6, and n-9 fatty acid-containing oils on CVD risks.

**Trial registration:**

ClinicalTrials.gov NCT01351012.

## Background

It is well established that decreasing dietary saturated fatty acids (SFA) reduces the risk of cardiovascular disease (CVD) [1]. Current dietary recommendations advise that unsaturated fatty acids should replace SFA with little guidance provided about the precise amounts that should be substituted [2]. Epidemiological evidence indicates various fatty acids classes including n-9 monounsaturated (MUFA), n-3 and n-6 polyunsaturated fatty acids (PUFA) as replacements for SFA with more health benefits [3-8]. Despite the large body of research evaluating the effects of different fatty acid classes, studies that systematically and simultaneously compare multiple fatty acid classes have not been conducted. Moreover, a need exists to evaluate additional biomarkers, beyond blood lipids and/or lipoproteins, that better estimate risk of clinical outcomes [9], and to achieve sufficiently large sample sizes in order to resolve modest differences and high variability in endpoint measurements. Additionally, significant knowledge gaps remain in our understanding of the effects of, and mechanisms underpinning the action of, the various fatty acid classes on risk factors for chronic diseases.

One such controversy is the debate surrounding α-linolenic acid (ALA). Whether its effects are dependent on its conversion to longer chain n-3 fatty acids [10-12] needs to be better substantiated. The relative efficacy of different classes of PUFA, specifically linoleic acid (LA) [13], ALA and docosahexaenoic acid (DHA), in modulating inflammatory processes and endothelial function also remains to be elucidated [14,15]. The reported undesirable effects of LA [16] are of particular concern. A direct comparison of dietary n-6 with n-3 fatty acids on inflammatory biomarkers and endothelial function would also be helpful in clarifying these issues. Since inflammation directly impacts endothelial function and the progression of atherosclerosis [17,18], endothelial function measurements would serve as useful biomarkers of CVD risk.

Abdominal obesity and insulin resistance are criteria for metabolic syndrome [19,20]. Dietary fat has been implicated in the pathogenesis of this syndrome via ectopic adipose tissue deposition [21,22]. Different classes of fatty acids appear to have different effects on body fat accretion [23-26]. Consequently, a need also exists to evaluate the effects of various fatty acid classes on body composition and body fat distribution.

Based on the above rationale, a multicenter randomized clinical trial was designed to evaluate the biological effects of conventional canola oil, high oleic canola oil, DHA-enriched high oleic canola oil, a blend of flax oil with safflower oil, and a blend of corn oil and safflower oil. The contrast selected allowed for comparisons of the effects of oils rich in n-9 versus n-3 and n-6 oil blends. Circulating lipids and/or lipoproteins, inflammatory biomarkers, endothelial function and body composition were evaluated after each dietary treatment period. Additionally, mechanistic assessments including reverse cholesterol transport, stable isotope fatty acid conversion, and fatty acid desaturase genetic variation analysis were also carried out. Consequently, the purpose of this study was to comprehensively investigate the effects of major fatty acid classes on biomarkers of chronic disease at a mechanistic level. This paper describes the protocol and subject recruitment experience for the COMIT study and presents plasma fatty acid data.

## Methods

### Study design

COMIT was designed as a five-phase randomized controlled double-blind crossover clinical trial. One-hundred and seventy subjects (n = 95 females) aged 18 years or older were selected with at least one metabolic syndrome clinical criterion. Participants were recruited at two participating centers in Canada, including the Richardson Centre for Functional Foods and Nutraceuticals (RCFFN) at the University of Manitoba in Winnipeg (n = 69), and the Institute on Nutrition and Functional Foods (INAF) at Laval University in Québec City (n = 58), as well as one clinical institution in the United States: the Departments of Nutritional Sciences and Biobehavioral Health, Pennsylvania State University, University Park (n = 43). The Risk Factor Modification Centre (RFMC) at St. Michael’s Hospital in Toronto, Canada participated in the sample analyses.

### Study recruitment and retention

**Figure 1:**
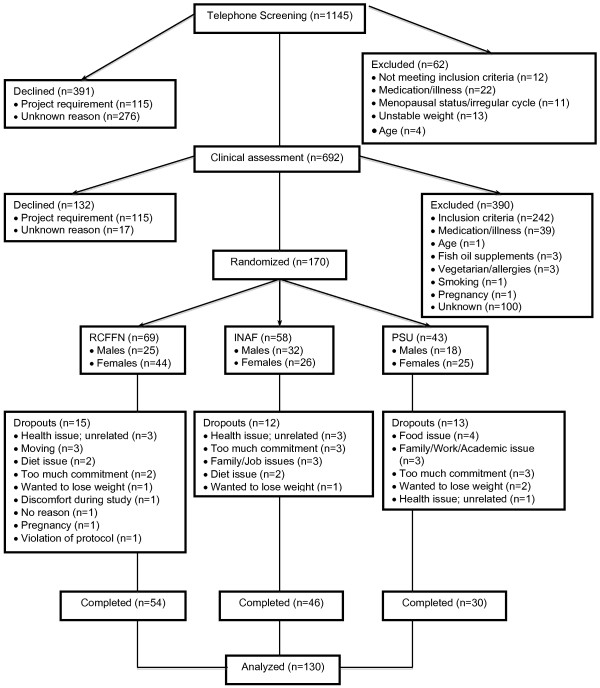
**Flow of participants in the COMIT study.** The participant flow through each step of the COMIT recruitment, screening and study protocol process. RCFFN, Richardson Centre for Functional Foods and Nutraceuticals. INAF, Institute on Nutrition and Functional Foods; PSU, Pennsylvania State University.

Recruitment was conducted using radio and newspaper advertisements, direct mailings, community meetings, and advertised information sessions as depicted in Table 1. On first contact, volunteers completed a questionnaire to determine their eligibility for the study. Qualifying participants were instructed to provide a fasting blood sample at their convenience. Eligible participants were selected based on the inclusion criteria and exclusion criteria described below. The flow of participants from initial contact to completion is given in Figure 1.

**Table 1:** Recruitment strategies employed at participating clinical sites

**Clinical sites**	**Recruitment tools**
**Winnipeg (Manitoba, Canada)**	Direct mail outs to neighborhoods in the City of Winnipeg
	University of Manitoba email system
	Radio advertisements
	Recruitment seminars at community centers at various localities
	University of Manitoba bulletin boards
**Québec City (Québec, Canada)**	Institute of Nutrition and Functional Foods (INAF) mailing list
	Laval University email system
	Newspaper advertisements
**University Park (Pennsylvania, United States)**	Pennsylvania State University email
	Flyers
	Local newspaper advertisements
	Television advertisements

At RCFFN in Winnipeg, a social program for study participants termed ‘Club Richardson’ was initiated for the purpose of fostering interaction and cooperation among participants to enhance their retention and compliance. Nutrition-related forums and outings were conducted, and tickets to hockey and soccer games were provided. Specific events were conducted to coincide with celebrations such as Christmas. Wine and cheese events were also held during the washout periods. Cards and flowers were gifted to coincide with birthdays. Similar retention strategies were used at the other two clinical centers. Personal interaction was a high priority among the clinical staff and study participants at all centers.

### Inclusion criteria

Initially, discussions about the eligibility criteria of the study were based on the following three parameters; 22 to 32 kg/m^2^ body mass index (BMI), plasma triglyceride >1.7 mmol/L, and waist to hip ratio of >0.85. When the protocol was finalized, it was decided that focus should be placed on waist circumference as the primary inclusion criterion because of the relationship between visceral fat and endothelial function [27]. At the beginning of the study, the Adult Treatment Panel III (ATP III) metabolic syndrome criteria for waist circumference (>102 cm for men and >88 cm for women) were followed [28]. As the trial progressed, the International Diabetes Federation (IDF) metabolic syndrome criteria for waist circumference (≥94 cm for men and ≥80 cm for women) were adopted [29] to identify individuals in the initial stages of abdominal obesity who might benefit from dietary intervention. This change occurred at the same time in all centers and did not affect the subjects already recruited as the waste circumference cutoff values were lowered. Other inclusion criteria were triglyceride level (TG) ≥1.7 mmol/L, high density lipoprotein cholesterol level (HDL) <1 mmol/L (males) or <1.3 mmol/L (females), blood pressure ≥130 mmHg (systolic) and/or ≥85 mmHg (diastolic) and glucose level ≥5.5 mmol/L. Participants were required to meet at least the waist criterion or any one of the other inclusion criteria in order to be eligible.

### Exclusion criteria

Individuals with thyroid disease (unless controlled by medication), diabetes mellitus, kidney disease, liver disease, current smokers, or those consuming more than two alcoholic drinks per week, were excluded from the study. Individuals taking medications known to affect lipid metabolism or endothelial function (including aspirin or other non-steroidal anti-inflammatory drugs), cholestyramine, colestipol, niacin, clofibrate, gemfibrozil, probucol, or 3-hydroxy-3-methyl-glutaryl-CoA (HMG-CoA) reductase inhibitors were excluded. In addition, individuals were not allowed to participate if they were unwilling to stop taking any supplements at least two-weeks prior to the study. Creatinine, serum glutamate pyruvate transaminase (SGPT), serum glutamic oxaloacetic transaminase (SGOT), alkaline phosphatase (ALKP), lactate dehydrogenase (LDH), glucose and lipid profiles that included total cholesterol (TC), TG, low density lipoprotein (LDL), and HDL were also analyzed at screening.

### Ethical considerations

The study protocols (with ethical considerations) were reviewed and approved by research ethics boards at each participating institution, including the Biomedical Ethics Board at the University of Manitoba, Comité sectoriel d’éthique de la recherche en sciences de la santé de l’Université Laval, and the Institutional Review Board at the Pennsylvania State University. The trial was registered at clinicaltrials.govunder the registration number NCT01351012. Written informed consent was obtained after an initial interview with each participant. The interview included a full description of the study and a discussion of the compliance issues and study expectations.

### Randomization

Random permutations of the five treatments were created by the study director at the main site using a random number generator to ensure that all the subjects received treatments in a balanced and random order. Sequences were coded for the purpose of blinding the investigators and the subjects. Coded sequences were kept in sealed envelopes at the Winnipeg center and were assigned to subjects by study coordinators at respective sites as they joined the study across the three centers.

### Study diets

Participants consumed a controlled weight-maintenance, full-feeding diet with a fixed macronutrient composition (35% fat, 50% carbohydrate, 15% protein, <200 mg cholesterol, 35 to 40 g fiber) for five treatment periods. They were provided with three meals and two snacks a day using a seven day rotating menu for the complete duration of each dietary phase. Each of five treatment phase extended four 4 weeks and separated by four-week washout intervals, although ten volunteers took shorter washout periods of two to four weeks at the PSU site. Treatment oils were provided as a shake-style beverage, divided in two equal doses to be consumed at breakfast and supper. Participants were instructed to consume one of their meals under supervision on site. Treatment oils were: 1) conventional canola oil (Canola), 2) high oleic canola oil (CanolaOleic), 3) 85% high oleic canola and 15% DHA oil blend (CanolaDHA), 4) 60% flax oil and 40% safflower oil blend (FlaxSaff), and 5) 25% corn oil and 75% safflower oil blend (CornSaff). The amount of treatment oil and/or oil blend was determined based on the calculated energy requirements of the participants, with the 3000 Kcal intake targeted to consume 60 g oil per day, and the 1800 and 2400 Kcal groups to consume 36 and 48 g per day, respectively. Oils were weighed to the nearest gram and added to the shakes. Meals and shakes were prepared in the metabolic kitchen where kitchen staff and clinical coordinators were blinded to the treatments. Meals and shakes were provided in cold packs for meals consumed offsite. These take-out meals were provided to participants at each center during their daily visit. Weekend meals and shakes were delivered to the participants’ place of residence or were dispensed at the feeding site on Fridays.

A subgroup of participants in Winnipeg prepared their own meals under the supervision of a trained individual. This was possible due to personal cultural practices involving the use of a common kitchen to prepare community meals. Treatment shakes were prepared at the RCFFN and delivered to the community every two to three days.

Subjects were instructed to consume only the prepared meals and to limit their consumption of alcohol or caffeinated beverages. Diets were planned for every subject according to their energy requirements and were nutritionally adequate. Energy requirement was calculated using the Mifflin-St Jeor equation [30]. During each study period body weights were monitored, and if subjects gained or lost weight energy adjustments were made. Subjects were given a diet that met the Canadian and United States recommended nutrient intakes. The nutrient content of the diet was analyzed using FOOD PROCESSOR (ESHA Research, Salem, Oregon, United States) to verify macronutrient composition and assure micronutrient adequacy.

### Compliance

Compliance during the trial was determined based on the remaining amounts of treatment shakes in pre-packaged food packs intended for off-site consumption, and by a weekly dietary questionnaire on dietary deviations which ensured that macro- and micronutrient intakes remained unchanged. Subjects were instructed to consume at least one meal on site, further ensuring compliance. These observations were further confirmed by expected changes in plasma fatty acid profiles at the end of each dietary period. For example, a greater than two-fold baseline to endpoint increase in serum DHA was expected during the CanolaDHA dietary period.

### Intervention

Four oils with different n-9: n-6: n-3 ratios were selected as the intervention; conventional canola oil (6:2:1), high oleic canola oil (31:6:1), DHA-enriched high oleic canola oil (6:1:1), and a flax oil and safflower oil blend (1:1:1). The fatty acid composition varied noticeably among treatment oils (Table 2), allowing comparison of the effects of n-3 fatty acids, ALA and DHA, n-6 fatty acid, LA, and n-9 fatty acid, oleic acid.

**Table 2:** **Fatty acid composition of dietary oils (%)**^a^

**Fatty acids**	**Canola**	**CanolaOleic**	**CanolaDHA (85:15)**	**FlaxSaff (60:40)**	**CornSaff (25:75)**
**c4:0**	0	0	0	0	0
**c6:0**	0	0	0	0	0
**c8:0**	0	0	0	0	0
**c10:0**	0	0	0	0	0
**c12:0**	0.09	0.06	0.07	0	0
**c14:0**	0.07	0.07	0.78	0	0.01
**c16:0**	4.06	3.66	5.25	4.90	5.86
**c17:0**	0.07	0.09	0.17	0	0.02
**c18:0**	1.83	1.83	1.70	3.17	1.90
**c20:0**	0.65	0.65	0.57	0	0.11
**C22:0**	0.31	0.31	0.30	0	0
**c24:0**	0.18	0.18	0.15	0	0
**Total SFA**	7.26	6.85	8.99	8.07	7.90
**c14:1**	0	0	0.01	0	0
**c16:1**	0.25	0.22	0.21	0	0.03
**c17:1**	0.12	0.20	0.17	0	0
**c18:1**	58.61	71.47	63.25	17.86	17.60
**c20:1**	1.22	1.20	1.03	0	0.03
**c22:1**	0.05	0.07	0.06	0	0
**Total MUFA**	60.25	73.16	64.73	17.86	17.66
**c18:2 n-6**	19.54	14.74	12.74	37.47	69.34
**c18:3 n-3**	9.76	2.30	1.96	31.98	0.29
**c20:4 n-6**	0	0	0.06	0	0
**c20:5 n-3**	0	0	0.15	0	0
**c22:5 n-3**	0	0	2.36	0	0
**c22:6 n-3**	0	0	5.80	0	0
**Total PUFA**	29.30	17.04	23.07	69.45	69.63
**Total n-6 PUFA**	19.54	14.74	12.80	37.47	69.34
**Total n-3 PUFA**	9.76	2.30	10.27	31.98	0.29

^a^The values are % of total fatty acids. The daily 3000 kcal diet block contained 60 g of treatment oil. Canola, conventional canola oil; CanolaOleic, high oleic canola oil; CanolaDHA, high oleic canola and DHA oil blend; FlaxSaff, flax and safflower oil blend; CornSaff, corn and safflower oil blend; SFA, saturated fatty acids; MUFA, monounsaturated fatty acids; PUFA, polyunsaturated fatty acids.

### Comparison dietary fat blend

An oil blend of corn (25%) and safflower (75%) with n-9: n-6: n-3 ratio of 6:25:0.1 was used as the comparison instead of a high saturated fat blend. Since the effects of saturated fat have been well established it was deemed unnecessary to include it as a control. Moreover, we previously examined the effects of high oleic canola and flax oil compared to a diet high in saturated fat [23,31]. Additionally n-6 fatty acids, which are abundant in the comparison oil blend, are considered essential and healthy, however the concept of the n-6 to n-3 ratio and the effects on inflammation merits re-examination [13,16]. Using n-6 rich oil enabled us to investigate these concepts.

### Outcome measures

Endothelial function was identified as the primary endpoint based on our rationale of evaluating emerging CVD risk factors beyond traditional lipid biomarkers for CVD. Secondary endpoints and laboratory analyses are presented in “List of secondary endpoints analyzed in the COMIT study” section. All centers followed identical procedures on all the measurements to minimize the variation. The peripheral arterial tonometry (PAT) technique was utilized to measure endothelial function. By using an Endo-PAT device (Itamar Medical, Caesarea, Israel), this technique measures the reactive hyperemia which is significantly correlated with endothelium-dependent vasodilatation [32]. Endothelial function testing was carried out after a 12 hour fast in the morning before breakfast at beginning and end of each phase. Scheduling pre-menopausal women for testing within the first seven days of their cycle was attempted to avoid the impact of hormonal fluctuations on the endothelial function test. Dual energy x-ray absorptiometry (DEXA) scanning and blood pressure recording using a digital blood pressure monitor were completed at beginning and end of each treatment. Blood pressure was recorded three times and the mean value used for data analysis. Waist circumference also was taken at the beginning and end of each phase. Twelve-hour fasting blood samples were collected on days 1, 2, 29 and 30 in both EDTA or heparin coated tubes. Blood samples obtained on days 1 and 2 were used to determine baseline values for study endpoints, whereas blood samples obtained on the two last days were used for the endpoint values. Blood was centrifuged for 20 minutes at 520 × g to separate either serum or plasma depending on the requirement of the analysis. Aliquots of plasma and sera were stored at −80°C until analysis. Deuterium oxide water was administered on day 29 (0.7 g × kg body water) to track ALA interconversion to longer chain n-3 PUFA.

List of secondary endpoints analyzed in the COMIT study

Secondary endpoints

Cholesterol

● **LDL**

● **HDL**

● **Triglycerides**

● **Blood glucose**

● **Apolipoprotein AI**

● **Apolipoprotein B**

● **CRP**

● **IL-6**

● **E-selectin**

● **ICAM-1**

● **VCAM-1**

● **Body fat%**

● **Lean mass**

● **Fat mass**

● **Android to gynecoid ratio**

● **Conversion of ALA to EPA and DHA**

● **Fatty acid ethanolamine metabolites of oleic acid**

● **FADS and ELOVL genetic variant (SNP) analyses**

● **Lipoprotein particle size**

● **Lipoprotein cholesterol ester composition**

● **Body weight**

● **Waist circumference**

● **Blood pressure**

LDL, low density lipoprotein; HDL, high density lipoprotein; CRP, C-reactive protein; IL-6, interleukin-6; ICAM-1, intercellular adhesion molecule-1; VCAM-1, vascular cell adhesion molecule-1; ALA, α-linolenic acid; EPA, eicosapentaenoic acid; DHA, docosahexaenoic acid; FADS, fatty acid desaturase; ELOVL, elongases of very long chain fatty acids; SNP, single nucleotide polymorphism.

Aliquots of 1 mm plasma EDTA samples obtained from all participants on days 29 and 30 of each phase were assembled at RCFFN for total plasma fatty acid profile analyses. Given the double-blinded crossover design, compliance was assessed by total plasma fatty acid concentrations across the three clinical sites and all five phases. Plasma fatty acids were extracted by the classic Folch method using chloroform-methanol (2:1, volume to volume (v/v)) [33] containing 0.01% butylated hydroxytoluene (Sigma-Aldrich, Oakville, Ontario, Canada), followed by methylation with methanolic HCl. Fatty acid methyl esters were then analyzed using an Agilent 6890 N gas chromatograph equipped with a flame ionization detector (Agilent Technologies, Mississauga, Ontario, Canada). During the extraction and methylation, heptadecanoic acid (C17:0) as an internal standard (Sigma-Aldrich, Oakville, Ontario, Canada). Known fatty acid standards (GLC-461, NuChek Prep, Inc. Elysian, Minnesota, United States) were used to identify the individual fatty acids in plasma samples. The level of each fatty acid was then calculated according to the corresponding peak area relative to the total area of total interested fatty acids, and considered as a percentage of the total fatty acids.

### Data management and statistical analysis

A sample size of 40 subjects per each of the three centers (120 in total) was determined based on endothelial function as the primary outcome. Endothelial function was intended to be assessed by flow mediated dilatation (FMD) The anticipated difference in means was 20% and the standard deviation was 1.35 [34]. Aforementioned sample size was sufficient to detect this difference with a power of 0.8 at a significance of 0.05. Endothelial function is a continuous variable which measures endothelium-dependent vasodilatation. Just before the beginning of the trial, it was decided to use the Endo-PAT technique for the measurement of the endothelial function, and a new sample size calculation was not made since reactive hyperemia index (RHI) measured by the Endo-PAT technique is significantly correlated with FMD [35] and has a similar variability [36]. We hypothesized that the favorable lipid changes associated with our proposed dietary interventions would improve endothelial function short-term. One hundred and seventy subjects were recruited with the anticipation of approximately a 20% dropout rate, based on our prior experience and considering the long-term commitment needed for the trial. Data from all centers were uploaded to a secure file storage system and specific centers were selected for analysis based on their expertise. Data were manually checked for accuracy. A per protocol approach with a mixed model analysis was decided as appropriate for data analysis utilizing SAS version 9.2. Kolmogorov-Smirnov (SAS version 9.2) or Cramer-von Mises (SAS version 9.2) tests and histograms were used to test the Gaussian nature of the variables and log transformation was used if necessary.

## Results

### Recruitment, screening, enrollment and retention

From the methodologies used to recruit participants (Table 1), the most successful recruitment strategy at the Winnipeg center was direct mailings in selected neighborhoods. Other centers used University email list-serves to recruit participants. Figure 1 presents the flow of participants through the study. After the initial contact, 63% of volunteers were screened for clinical assessment. Only 24% of the volunteers who underwent clinical assessment were eligible for the study. A total of 170 men and women were randomized, which represents 15% of the total number who underwent primary screening. The enrollment rate was 17%, 18% and 12% for the Winnipeg, Québec City and University Park centers, respectively.

As shown in Figure 1, Winnipeg had the highest number of participants (n = 69), while University Park had the fewest (n = 43). The dropout rates in the centers were 22%, 21%, and 30% for Winnipeg, Québec City and University Park, respectively. The overall dropout rate was 23.5%. The effect of the dropouts on the study was minimal because 77.5% of the dropouts occurred within the first two phases of the study. Moreover, baseline characteristics of the dropouts and the completers did not differ significantly (data not shown). Challenges in retaining participants were expected given the nine-month duration of the trial and participant study requirements (frequent visits to the centers, as well as the full-feeding protocol). As presented in Figure 1, health issues, moving out of town, level of commitment and job issues were the major factors behind cessation in the Winnipeg and Québec City centers. Food issues and academic or work issues were the major reasons for the University Park center.

### Participant characteristics

Participants were randomized to dietary treatments from September 2010 through to March 2012. Participants were classified as having metabolic syndrome as defined by IDF (Table 3). Winnipeg had the highest percentage of participants with metabolic syndrome (56%), followed by University Park (43%) and Québec City (39%). Overall, 47% of participants were characterized as having metabolic syndrome. Although almost 50% of the participants had metabolic syndrome, their 10-year coronary heart disease risk score was less than 4% as determined by Framingham risk score calculation (data not shown). Baseline characteristics of participants from the different centers and the entire cohort are presented in Table 4.

**Table 3:** **The number of metabolic syndrome criteria met by the participants in the COMIT study at each participating center**^**a**^

	**Winnipeg**	**Québec city**	**University Park**	**Total**
**One**	13.0%	6.5%	0%	7.7%
**Two**	31.5%	54.4%	56.7%	45.4%
**Three**	16.7%	26.1%	30.0%	23.1%
**Four**	33.3%	10.9%	13.3%	20.8%
**Five**	5.6%	2.2%	0%	3.1%

^a^Based on International Diabetic Federation defined criteria for metabolic syndrome; waist circumference (≥94 cm for men and ≥80 cm for women), triglycerides ≥1.7 mmol/L, high density lipoprotein <1 mmol/L (males) or <1.3 mmol/L (females), blood pressure ≥130 mmHg (systolic) and/or ≥85 mmHg (diastolic) and glucose ≥ 5.5 mmol/L.

**Table 4:** Baseline characteristics of participants at each participating center

	**Winnipeg**	**Québec city**	**University Park**	**Total**	***P *** **value**^**a**^
**Male**	16	29	15	60	
**Female**	38	17	15	70	0.003
**BMI (kg/m**^**2**^**)**	29.7 ± 5.0	30.0 ± 4.3	29.7 ± 3.3	29.8 ± 4.4	0.928
**Age (years)**	43.9 ± 15.8	49.9 ± 14.2	45.9 ± 9.6	46.5 ± 14.2	0.105
**Total cholesterol (mmol/L)**	5.4 ± 1.1	5.4 ± 1.0	5.1 ± 0.9	5.3 ± 1.1	0.371
**HDL (mmol/L) female**	1.3 ± 0.1	1.4 ± 0.1	1.3 ± 0.1	1.3 ± 0.1	0.413
**male**	1.1 ± 0.1	1.1 ± 0.1	1.1 ± 0.1	1.1 ± 0.1	0.906
**LDL (mmol/L)**	3.4 ± 1.0	3.4 ± 0.9	3.2 ± 0.9	3.4 ± 0.9	0.572
**Triglycerides (mmol/L)**	1.6 ± 0.9	1.8 ± 0.8	1.5 ± 0.9	1.7 ± 0.9	0.338
**Glucose (mmol/l)**	5.4 ± 1.7	5.3 ± 0.6	5.3 ± 0.5	5.4 ± 1.1	0.788
**Body weight (kg)**	80.7 ± 15.9	85.8 ± 15.3	89.9 ± 15.7	84.7 ± 15.9	0.042
**Waist (cm) female**	90.6 ± 11.9	97.4 ± 10.3	98.5 ± 9.9	93.4 ± 11.6	0.033
**male**	103.4 ± 13.8	106.9 ± 8.7	106.5 ± 6.5	105.8 ± 9.9	0.508
**SBP (mmHg)**	122.4 ± 20.3	119.8 ± 14.2	118.5 ± 12.4	120.6 ± 16.7	0.548
**DBP (mmHg)**	81.2 ± 12.4	70.5 ± 10.5	79.7 ± 7.40	77.0 ± 11.8	<0.001

^a^Analysis of variance was used to analyze between-group differences in continuous variables. For categorical variables, Fisher’s exact test or chi square test was used. BMI, body mass index; HDL, high density lipoprotein; LDL, low density lipoprotein; SBP, systolic blood pressure; DBP, diastolic blood pressure.

### Post-treatment fatty acid profiles

Total plasma fatty acid profiles of the completers of all five phases are summarized in Table 5. All fatty acid concentrations were calculated as percentage values of total identified fatty acids measured. As DHA was part of the dietary treatment, post-treatment plasma DHA values were used to evaluate the overall compliance of the intervention study. The observed significant increases in DHA concentration only following the CanolaDHA treatment indicated good compliance in all centers. As well as the shift in DHA levels, other changes also successfully reflected their dietary intake after the 28-day treatment phases. Briefly, Canola and CanolaOleic showed the highest level of MUFA contents (*P* < 0.05), since their oleic acid levels were significantly higher than other treatments. Two PUFA-rich diets significantly elevated PUFA contents compared to the other three canola-based MUFA diets (*P* < 0.05), due to their higher levels of linoleic acid. FlaxSaff provided the lowest level of arachidonic acid (*P* < 0.05), indicating the competition between n-3 and n-6 synthesis as the FlaxSaff oil treatment provided the highest level of dietary ALA. CanolaDHA and FlaxSaff also showed higher levels of EPA compared to other three treatments (*P* < 0.05). In general, the ratio of n-6 to n-3 also successfully explained the corresponding response to n-3 rich and n-6 rich dietary treatments, indicating good compliance among participants across the entire study.

**Table 5:** **Plasma fatty acid profile of participants at the end of each dietary phase in 130 participants (g/100 g)**^**a**^

	**Canola**	**CanolaOleic**	**CanolaDHA**	**FlaxSaff**	**CornSaff**
**c14:0**	0.71 ± 0.04	0.74 ± 0.04	0.71 ± 0.04	0.72 ± 0.04	0.68 ± 0.04
**c14:1**	0.12 ± 0.02	0.08 ± 0.02	0.09 ± 0.02	0.08 ± 0.02	0.07 ± 0.02
**c16:0**	27.04 ± 0.20^a^	27.40 ± 0.20^a^	28.10 ± 0.20^b^	27.41 ± 0.20^a^	27.35 ± 0.20^a^
**c16:1**	1.14 ± 0.08	1.10 ± 0.08	0.94 ± 0.08	1.30 ± 0.08	1.13 ± 0.75
**c18:0**	11.83 ± 0.10^a^	11.79 ± 0.10^a^	12.28 ± 0.10^b^	12.51 ± 0.10^c^	12.34 ± 0.10^bc^
**c18:1**	14.90 ± 0.19^a^	15.52 ± 0.19^b^	13.36 ± 0.19^c^	12.10 ± 0.18^d^	11.62 ± 0.18^d^
**c18:2 n-6**	22.00 ± 0.23^a^	21.52 ± 0.23^a^	18.68 ± 0.23^b^	25.13 ± 0.23^c^	25.93 ± 0.23^d^
**c18:3 n-6**	0.17 ± 0.07	0.18 ± 0.07	0.23 ± 0.07	0.12 ± 0.07	0.17 ± 0.07
**c18:3 n-3**	0.79 ± 0.03^a^	0.63 ± 0.03^b^	0.57 ± 0.03^bc^	1.61 ± 0.03^c^	0.49 ± 0.03^d^
**c20:0**	0.50 ± 0.02^a^	0.48 ± 0.02^ab^	0.49 ± 0.02^a^	0.41 ± 0.02^b^	0.46 ± 0.02^ab^
**c20:1**	0.32 ± 0.02^a^	0.32 ± 0.02^a^	0.27 ± 0.02^ab^	0.18 ± 0.02^c^	0.23 ± 0.02^bc^
**c20:2 n-6**	0.35 ± 0.03^a^	0.31 ± 0.03^ab^	0.24 ± 0.03^b^	0.32 ± 0.03^ab^	0.37 ± 0.03^a^
**c20:3 n-6**	2.45 ± 0.07^ab^	2.53 ± 0.07^b^	1.79 ± 0.07^c^	1.86 ± 0.07^c^	2.28 ± 0.07^a^
**c20:4 n-6**	9.28 ± 0.15^a^	9.67 ± 0.15^b^	9.70 ± 0.15^b^	8.27 ± 0.15^c^	9.59 ± 0.15^ab^
**c20:5 n-3**	1.09 ± 0.04^a^	0.86 ± 0.04^b^	1.53 ± 0.04^c^	1.45 ± 0.04^c^	0.49 ± 0.04^d^
**c22:0**	0.94 ± 0.03^ab^	0.90 ± 0.03^ab^	1.00 ± 0.03^a^	0.90 ± 0.03^b^	0.93 ± 0.03^b^
**c22:4 n-6**	0.43 ± 0.08	0.29 ± 0.08	0.25 ± 0.08	0.22 ± 0.08	0.39 ± 0.08
**c22:5 n-3**	0.81 ± 0.03^a^	0.72 ± 0.03^b^	0.34 ± 0.03^c^	0.97 ± 0.03^d^	0.62 ± 0.03^e^
**c22:6 n-3**	2.84 ± 0.09^a^	2.79 ± 0.09^a^	7.21 ± 0.10^b^	2.59 ± 0.09^a^	2.66 ± 0.09^a^
**c24:0**	0.72 ± 0.03^a^	0.65 ± 0.03^b^	0.74 ± 0.03^a^	0.74 ± 0.03^a^	0.73 ± 0.03^a^
**c24:1**	1.56 ± 0.05^a^	1.49 ± 0.05^ab^	1.55 ± 0.05^a^	1.38 ± 0.05^b^	1.44 ± 0.05^ab^
**Total SFA**	41.74 ± 0.19^a^	41.92 ± 0.19^ab^	43.28 ± 0.19^c^	42.71 ± 0.19^cd^	42.51 ± 0.19^bd^
**Total MUFA**	18.05 ± 0.22^a^	18.50 ± 0.22^a^	16.20 ± 0.22^b^	14.78 ± 0.22^c^	14.49 ± 0.22^c^
**Total PUFA**	40.21 ± 0.22^a^	39.54 ± 0.22^b^	40.47 ± 0.22^a^	42.57 ± 0.22^c^	43.04 ± 0.22^c^
**Total n-6 PUFA**	34.67 ± 0.23^a^	34.46 ± 0.23^a^	30.92 ± 0.23^b^	35.92 ± 0.23^c^	38.79 ± 0.23^d^
**Total n-3 PUFA**	5.53 ± 0.10^a^	5.03 ± 0.10^b^	9.62 ± 0.11^c^	6.64 ± 0.10^d^	4.24 ± 0.10^e^
**n-6:n-3**	6.55 ± 0.13^a^	7.18 ± 0.14^b^	3.32 ± 0.14^c^	5.65 ± 0.13^d^	9.41 ± 0.13^e^

^a^The values are % abundance of each fatty acid to total fatty acids given as least squares mean ± SE for 130 individuals. Total fatty acid values and ratios are calculated. Mixed-effects repeated-measures analysis of variance with treatment, age, gender and the dietary phase as fixed effect, the last diet (sequence effect) as a random effect, and the measures for each subject by phases were used for the data analysis. Canola, conventional canola oil; CanolaOleic, high oleic canola oil; CanolaDHA, high oleic canola and DHA oil blend; FlaxSaff, flax and safflower oil blend; CornSaff, corn and safflower oil blend; SFA, saturated fatty acids; MUFA, monounsaturated fatty acids; PUFA, polyunsaturated fatty acids. ^abcde^Mean values with different superscript letters across rows denote significant differences at *P* < 0.05.

### Primary and secondary outcome measures

Data analyses of endothelial function and secondary outcome measures given in “List of secondary endpoints analyzed in the COMIT study” section are currently being carried out.

### Adverse effects

Adverse effects were monitored using a questionnaire distributed by study staff on every Friday of each dietary phase. Additionally, the clinical study staff assessed the tolerability of the treatment each day when they interacted with the participants. No adverse effects of any treatment diets were reported.

## Discussion

The recruitment and retention strategies employed resulted in the successful completion of the required number of participants. Retention was also high due to the dedication and commitment of the study volunteers and clinical coordinators, as well as the success of the social retention enhancement program. Differences across centers in demographic characteristics and baseline parameters were limited to the male to female ratio, body weight, waist circumference in females, and diastolic blood pressure. The success rate of participant completion was numerically close between Winnipeg and Québec City, with the University Park center having a lower rate. The major reason for dropouts in Winnipeg and Québec City were health reasons, while in University Park it was more related to experimental diet acceptability.

Predictable elevations of specific plasma fatty acids representing the major dietary fatty acids in the intervention oils were observed as expected. For example, the three-fold elevation of plasma ALA following the FlaxSaff dietary phase compared to the CornSaff dietary phase demonstrated the ability of flax oil-enriched shakes to raise serum ALA levels. Similarly, the 1.3-fold elevation of oleic acid after the CanolaOleic treatment compared to CornSaff treatment is consistent with its high oleic acid content. Significantly higher eicosapentaenoic acid (EPA) levels in Canola and FlaxSaff compared to other treatments are evidence for the metabolic conversion of ALA to EPA. The significant elevation of EPA in CanolaDHA compared to other treatments (except FlaxSaff) could probably be due to the higher EPA content in the algal sourced DHA that was used in this study. However DHA might convert back to EPA in CanolaDHA, which can be further confirmed by the stable isotope-trafficking assays in this study [37,38]. Similarly, the more than two-fold elevation of DHA after the CanolaDHA treatment, compared to other treatments, is not surprising and is in line with previous reports [39-41]. Lack of a DHA raising effect of FlaxSaff treatment agrees with prior work demonstrating low efficiency of conversion of ALA to DHA [31]. Remarkable consistency was observed in compliance among participants as depicted here by predictable shifts in their fatty acid profile at the end of each dietary period, highlighting the successful completion of this long-term full-feeding clinical trial.

Multicenter randomized controlled clinical trials exist as the gold standard for evidence-based research [42]. The multicenter nature of the trial ensured the robustness of results across different centers. The iso-caloric, weight-maintaining, full-feeding regimen eliminated the effect of variable habitual, background diets on study outcomes. Caloric excess or restriction can potentially affect blood lipids, and even body composition and body weight loss can affect endothelial function [27], hence requiring standardized study conditions to prevent weight change. Study treatments oils were blended at the RCFFN and shipped to participating centers, removing an important source of variation. Sample analysis for specific endpoints was completed for all samples at the center designated for a particular analysis, in order to remove the effect of inter-laboratory variation. Clinical measurement protocols, particularly for endothelial function testing, were harmonized across centers by training personnel from the University Park center staff. The crossover design eliminated the effect of individual differences on response to treatments and reduced the required sample size. Endothelial function was found to be significantly changed in studies with a crossover or a parallel design over a period of four weeks following dietary intervention, indicating that the study design and the duration of the current study is appropriate for detecting changes in endothelial function [43,44]. Moreover, an acute study that measured endothelial function by FMD four hours after a high-fat meal supplemented with either 25 g olive oil or 40 g of shelled walnut in hypercholesterolemic subjects [34] also showed significant changes. FMD [45] and Endo-PAT techniques [46] are both dependent on nitric oxide mediated vasodilatation which is affected by both short- and long-term changes in endothelial function. Even though the measure of percent change in endothelial function has no clinical relevance as yet, a lower range of endothelial function (lower two tertiles) as measured by FMD, is associated with enhanced risk of future cardiovascular events [47].

The final sample size of 130 allowed us to detect a difference of 9.4% or greater in the RHI, which is a measure of the endothelial function, and a 10% difference of LDL-C with a power of 0.8 at a significance of 0.05.

Diets were designed to address contemporary nutrition questions. Although MUFA generally decreases TC and LDL-C, the efficacy is considered lesser than PUFA [48,49]. However, the relative benefits of individual unsaturated fatty acid classes are increasingly being challenged; this study was designed to resolve some of these issues.

The primary and ancillary measurements in our study will link the changes in surrogate cardiovascular biomarkers such as blood lipids and glucose, inflammatory biomarkers and hormones, to clinical endpoints such as endothelial function and body fat composition. The mechanistic studies, including lipid trafficking, apolipoproteins, lipoprotein particle size, and reverse cholesterol transport, will assist in clarifying the relationship between the biomarkers and clinical endpoints. Finally, results will be evaluated from the perspective of genetic variation, assessed through single nucleotide polymorphisms among individuals to assess why biomarker responses vary as a function of diet treatments.

The study possesses certain limitations. The disparity in male: female ratio and the baseline differences in cholesterol across centers can be considered as confounding treatment effects. However, use of a mixed model approach in statistical analyses is expected to minimize this effect. The effect of the preceding diet or the sequence of treatment is another confounding factor that was anticipated to be minimized by the mixed model approach. Moreover, differences in bioactives other than fatty acid composition within the treatment oils might affect clinical measurements such as endothelial function. The intra-individual and inter-operator variability in endothelial function testing might likewise obscure a treatment effect. Thus, standardization of the test procedure represents an important factor in maintaining the robustness of endothelial function data [50]. Steps were taken to limit this variability by controlling the environment and also by minimizing any additional factors such as the stage in the menstrual cycle of female participants [51], medication, and tea or coffee drinking [52]. Additionally, use of the corn and safflower oil blend as the alternate treatment would be expected to improve blood lipids and other parameters [6]. Therefore, it may not be possible to see an improvement in blood lipids in certain treatments compared to the corn and safflower oil blend treatment.

## Conclusions

The successful completion of 130 participants through the study protocol of this nine-month long, full-feeding, multicenter study represents a significant achievement. Recruitment and retention strategies used in this study were instrumental in achieving this goal. Standardized protocols and efforts to minimize variation are expected to lead to findings that will help to fill the gaps in our knowledge of the metabolism, lipid trafficking, and clinical efficacy of the major classes of dietary fatty acids.

### Trial status

The trial started recruiting volunteers in September 2010 and completed the intervention stage in March 2012.

## Competing interests

PJJ reported receiving grants from Advanced Foods and Materials Network (AFM Net), Danone, Enzymotec, Unilever, the Canadian Institutes of Health Research (CIHR) and Canada Research Chair Endowment (CRCE) of the Federal Government of Canada. PJJ also serves as President of Nutritional Fundamentals for Health Inc, which markets plant sterols among other nutraceuticals. BL has received research grants from the Dairy Farmers of Canada, Dairy Australia, Danone Institute and Atrium Innovations, and honoraria from Unilever, Danone, and the Dairy Farmers of Canada. BL is Chair in Nutrition and Cardiovascular Health, supported in part by Provigo/Loblaws. DAJ reported serving on the Scientific Advisory Board of Unilever, Sanitarium Company, California Strawberry Commission, Loblaw Supermarket, Herbal Life International, Nutritional Fundamental for Health, Pacific Health Laboratories, Metagenics, Bayer Consumer Care, Orafti, Dean Foods, Kellogg’s, Quaker Oats, Procter & Gamble, Coca-Cola, NuVal Griffin Hospital, Abbott, Pulse Canada, Saskatchewan Pulse Growers, and Canola Council of Canada; receiving honoraria for scientific advice from the Almond Board of California, International Tree Nut Council Nutrition Research and Education Foundation, Barilla, Unilever Canada, Solae, Oldways, Kellogg’s, Quaker Oats, Procter & Gamble, Coca-Cola, NuVal Griffin Hospital, Abbott, Canola Council of Canada, Dean Foods, California Strawberry Commission, Haine Celestial, and Alpro Foundation; being on the speakers panel for the Almond Board of California; receiving research grants from Loblaw Brands Ltd, Unilever, Barilla, Almond Board of California, Solae, Haine Celestial, Sanitarium Company, Orafti, International Tree Nut Council, and Peanut Institute; and receiving travel support to meetings from the Almond Board of California, Unilever, Alpro Foundation, and International Tree Nut Council, Canadian Institutes for Health Research, Canada Foundation for Innovation, Ontario Research Fund. DAJ receives salary support as a Canada Research Chair from the federal government of Canada. DAJ’s wife is a director of Glycemic Index Laboratories, Toronto, Ontario, Canada. SGW has received research funding and consulting fees from the Canola Council of Canada and Flax Canada 2013. PMKE serves on the Unilever Scientific Advisory Board. Other authors did not report any conflicts of financial interests.

## Authors’ contributions

PJJ is responsible for the integrity and the accuracy of the data and had full access to the complete data set in the study. Study concept and design: PJJ, DAJ, BL, SGW and PMKE. Acquisition of data: PJJ, DAJ, BL, SGW, PMKE, VKS, SP, JAF, XL and CEM, Analysis and interpretation of data: PJJ, VKS and SP. Drafting of the manuscript: VKS, SP, and PJJ. Critical revision of the manuscript for important intellectual content and final approval of the manuscript: PJJ, DAJ, BL, SGW and PMKE. Obtained funding: PJJ, DAJ, BL, SGW and PMKE. Study supervision: PJJ, DAJ, BL, SGW and PMKE. All authors reviewed the manuscript. All authors read and approved the final manuscript.
